# Corrigendum to “Degradation of Epidermal Growth Factor Receptor Mediates Dasatinib-Induced Apoptosis in Head and Neck Squamous Cell Carcinoma Cells” [*Neoplasia* 14 (2012) 463–475]

**DOI:** 10.1016/j.neo.2016.12.013

**Published:** 2017-02-28

**Authors:** Yu-Chin Lin, Meng-Hsuan Wu, Tzu-Tang Wei, Shu-Hui Chuang, Kuen-Feng Chen, Ann-Lii Cheng, Ching-Chow Chen

**Affiliations:** * Graduate Institute of Pharmacology, National Taiwan University College of Medicine, Taipei, Taiwan; † Department of Oncology, National Taiwan University Hospital, Taipei, Taiwan; ‡ Division of Oncology and Hematology, Department of Internal Medicine, Far-Eastern Memorial Hospital, Taipei, Taiwan; § National Center of Excellence for Clinical Trial and Research, National Taiwan University Hospital, Taipei, Taiwan; ¶ Medical Research, National Taiwan University Hospital, Taipei, Taiwan; # Department of Internal Medicine, National Taiwan University Hospital, Taipei, Taiwan

In Figure 2, the Src band in the Western blot of FaDu was wrongly placed and we have revised it (arrow) in the new Figure 2 (below).

**Figure 2:**
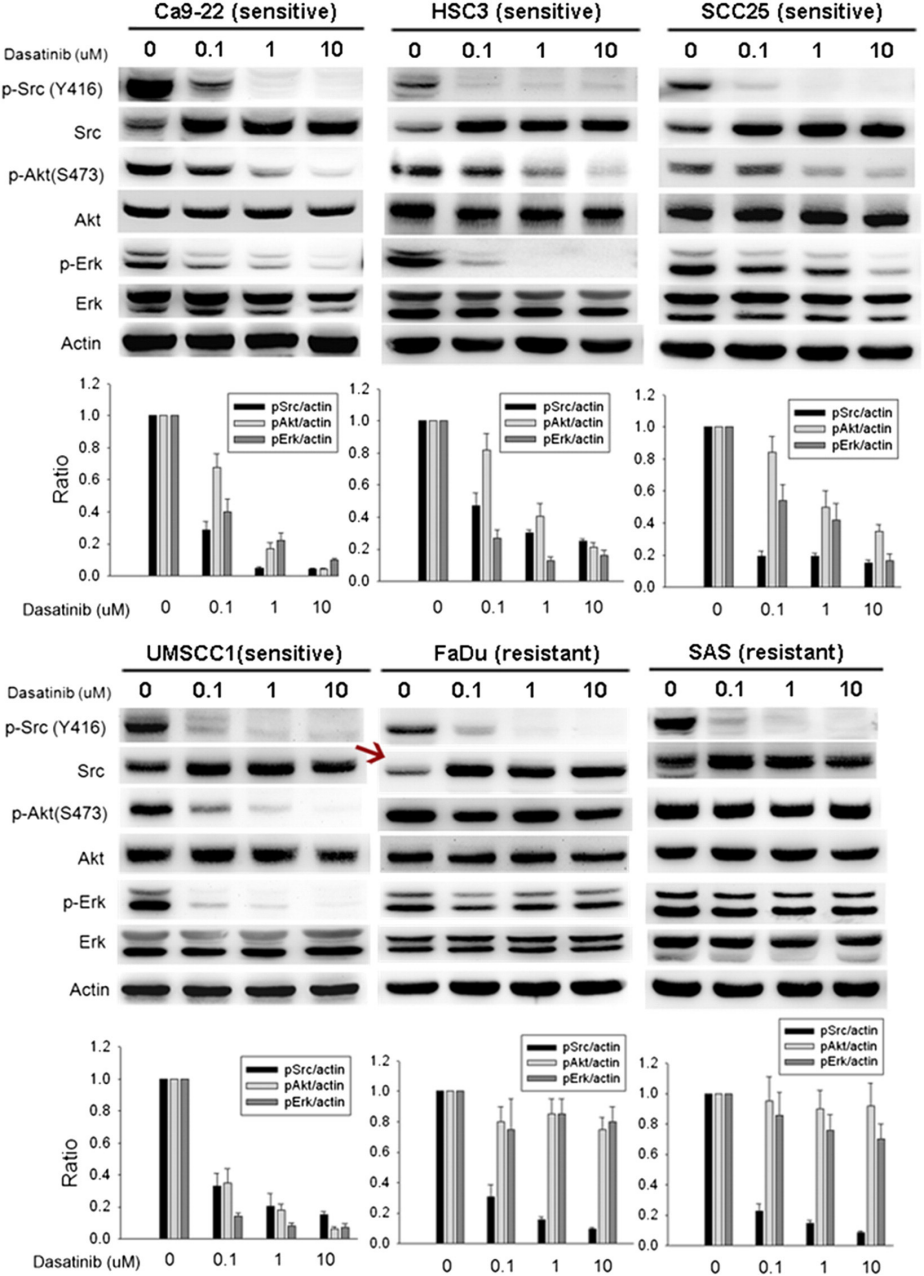
Effect of dasatinib on Src, Akt, Erk, and Bcl-2.

In Figure 5*A*, the expressions of p-EGFR, EGFR, and p-Erk in HSC3 cells were wrongly placed and we have revised them (arrows) in the new Figure 5 (below).

**Figure 5:**
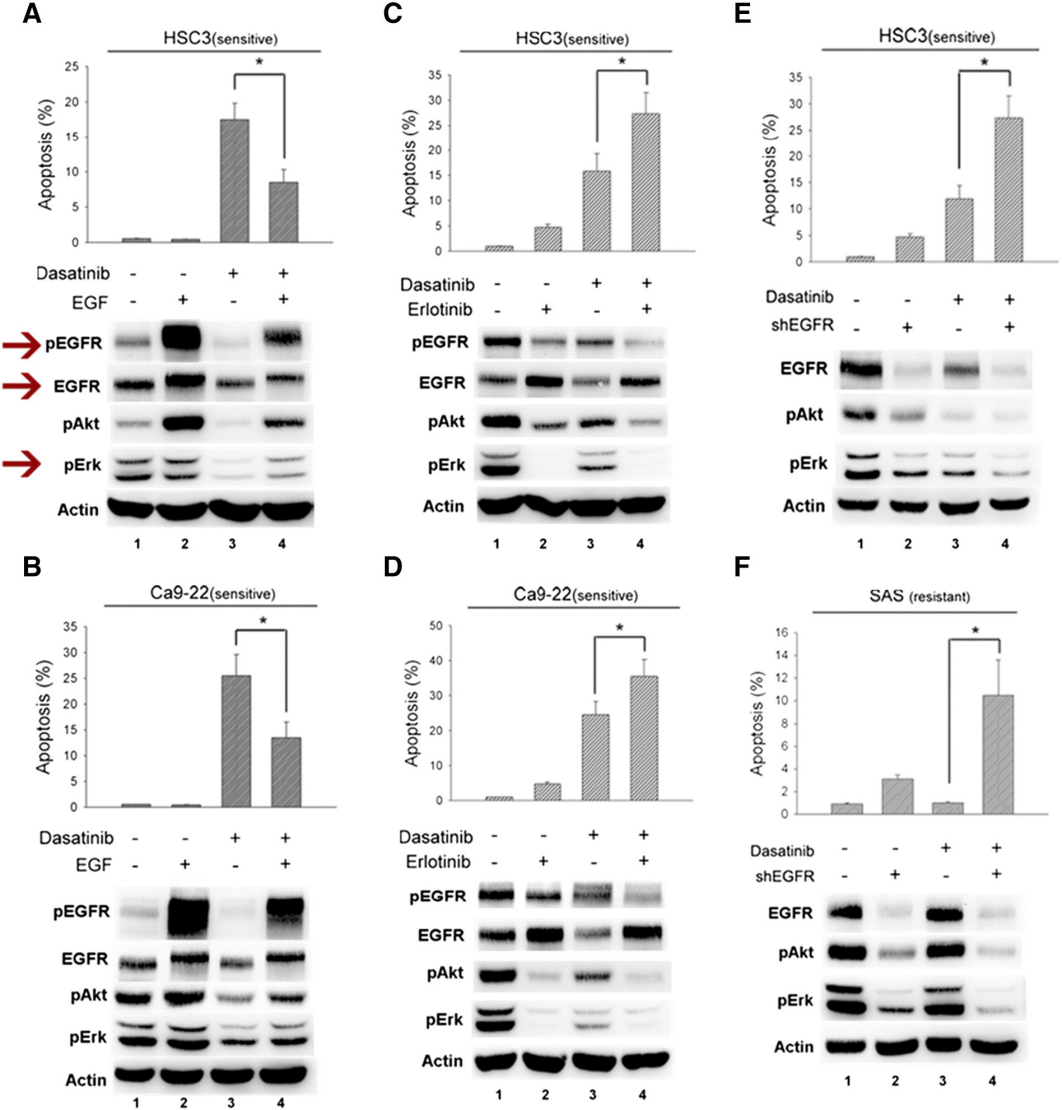
Role of EGFR in dasatinib-induced apoptosis.

